# The Dissemination and Molecular Characterization of Clonal Complex 361 (CC361) Methicillin-Resistant *Staphylococcus aureus* (MRSA) in Kuwait Hospitals

**DOI:** 10.3389/fmicb.2021.658772

**Published:** 2021-05-06

**Authors:** Eiman Sarkhoo, Edet E. Udo, Samar S. Boswihi, Stefan Monecke, Elke Mueller, Ralf Ehricht

**Affiliations:** ^1^Faculty of Medicine, Department of Microbiology, Kuwait University, Kuwait City, Kuwait; ^2^Leibniz Institute of Photonic Technology (IPHT), Jena, Germany; ^3^InfectoGnostics Research Campus Jena, Jena, Germany; ^4^Institute of Physical Chemistry, Friedrich Schiller University Jena, Jena, Germany

**Keywords:** CC361-MRSA, molecular typing, DNA microarray, MLST, spa typing

## Abstract

Methicillin-resistant *Staphylococcus aureus* (MRSA) belonging to clonal complex 361 (CC361-MRSA) is rare among patients’ populations globally. However, CC361-MRSA has been isolated with an increasing trend among patients in Kuwait hospitals since 2010. This study investigated the molecular characteristics of CC361-MRSA isolated from patients in Kuwait hospitals in 2016–2018 to understand their genetic relatedness and virulence determinants. Of 5,223 MRSA isolates investigated by DNA microarray, 182 (3.4%) isolates obtained in 2016 (*N* = 55), 2017 (*N* = 56), and 2018 (*N* = 71) were identified as CC361-MRSA. The CC361-MRSA isolates were analyzed further using antibiogram, *spa* typing and multi locus sequence typing (MLST). Most of the isolates were resistant to fusidic acid (64.8%), kanamycin (43.4%), erythromycin (36.3%), and clindamycin (14.3%) encoded by *fusC*, *aphA3*, and *erm(B)/erm(C)* respectively. Nine isolates (4.9%) were resistant to linezolid mediated by *cfr.* The isolates belonged to 22 *spa* types with t3841 (*N* = 113), t315 (*N* = 16), t1309 (*N* = 14), and t3175 (*N* = 5) constituting 81.3% of the *spa* types, four genotypes (strain types), CC361-MRSA-[V/VT + fus] (*N* = 112), CC361-MRSA-IV, WA MRSA-29 (*N* = 36), CC361-MRSA-V, WA MRSA-70/110 (*N* = 33) and CC361-MRSA-[V + fus] variant (*N* = 1). MLST conducted on 69 representative isolates yielded two sequence types: ST361 (11/69) and ST672 (58/69). All CC361-MRSA isolates were positive for *cap8*, *agr1*, and the enterotoxin *egc* gene cluster (*seg, sei, selm, seln, selo*, and *selu*). The *tst1* was detected in 19 isolates. The immune evasion cluster (IEC) genes type B (*scn*, *chp*, and *sak*) and type E (*scn* and *sak*) were detected in 20 and 152 isolates, respectively. The CC361-MRSA circulating in Kuwait hospitals consisted of two closely related sequence types, ST361 and ST672 with ST672-MRSA [V/VT + fus] as the dominant genotype. The dissemination of these newly emerged clones and the emergence of linezolid resistance limits therapeutic options, as well as present significant challenges for the control of MRSA infections in Kuwait hospitals.

## Introduction

Methicillin-resistant *Staphylococcus aureus* (MRSA) remains a major universal healthcare problem as it causes a wide range of infections including skin and soft tissue infections (SSTI), pneumonia, bacteremia, endocarditis, and osteomyelitis (McCaig et al., 2006; Hassoun et al., 2017). The epidemiology of MRSA has changed significantly since its description in the 1960s. MRSA has evolved from being an exclusive healthcare-acquired pathogen (healthcare-acquired MRSA (HA-MRSA) (McCaig et al., 2006), to being acquired outside the healthcare facilities in the communities (Community-associated MRSA, CA-MRSA) (Udo et al., 1993; McCaig et al., 2006; David and Daum, 2010). Recently, MRSA has also evolved to become a livestock-associated pathogen designated Livestock-associated MRSA (Armand-Lefevre et al., 2005; van Cleef et al., 2011). HA-MRSA are characteristically multi-resistant to antibiotics and carry SCC*mec* types I, II or III (Monecke et al., 2011). In contrast, CA-MRSA are usually more susceptible to non-beta-lactam antibiotics and carry SCC*mec* types IV, V, or VI (David and Daum, 2010). The Livestock-associated MRSA (LA-MRSA) initially caused major problems in agriculture and were the leading cause of bovine mastitis (Fluit, 2012) but have now become prominent among livestock, people associated with livestock and those with no previous contact with livestock (Graveland et al., 2011; Köck et al., 2013; Wagenaar et al., 2009; Boswihi et al., 2020a).

Molecular typing tools including staphylococcal protein A (spa) typing, multilocus sequence typing (MLST), pulsed-field gel electrophoresis, staphylococcal cassette chromosome mec (SCC*mec*) typing, DNA microarray, and whole genome sequencing (WGS) have been used in the epidemiologic surveillance of MRSA strains to detect and monitor emerging and reemerging infections as well as monitoring geographic spread and shifts of epidemic and endemic clones (Pfaller, 1999; van Belkum et al., 2001; Monecke et al., 2011; Boswihi et al., 2016, 2020a,2020b; Rebic et al., 2016). The application of these typing techniques to type MRSA from different geographic regions have shown that most of the MRSA infections reported worldwide were caused by a limited number of pandemic MRSA clones belonging to clonal complexes 5 (CC5), CC8/ST239, CC22, CC30, and CC45 (Robinson and Enright, 2003; Monecke et al., 2011; Guthrie et al., 2020) although CA-MRSA isolates belong to more diverse genetic backgrounds compared to HA-MRSA (Monecke et al., 2011; Udo, 2013; Tong et al., 2015; Guthrie et al., 2020).

Methicillin-resistant *Staphylococcus aureus* strains belonging to CC361 (CC361-MRSA) were described as rare and were reported sporadically in humans (Afroz et al., 2008; Weber et al., 2010; Coombs et al., 2011; Monecke et al., 2011; Shambat et al., 2012; Kinnevey et al., 2014), Rhesus monkeys (Roberts et al., 2019), Cattle (Tegegne et al., 2017), and in ready-to-eat food items (Islam et al., 2019). However, the CC361-MRSA have been increasingly isolated from human patients in the Arabian Gulf countries of Saudi Arabia (Senok et al., 2019), United Arab Emirates (Senok et al., 2020) and Kuwait (Boswihi et al., 2018, 2020a,2020b).

The first two known isolates of CC361-MRSA were isolated in Kuwait in 2010 from two patients in two different hospitals (Boswihi et al., 2016). Since then, the proportion of MRSA isolates belonging to CC361-MRSA has been increasing annually (Boswihi et al., 2018, 2020a,2020b). In this study, we investigated CC361-MRSA isolated from patients in public hospitals in Kuwait from 1 January, 2016 to 31 December, 2018, using staphylococcal protein A (*spa*) typing, multi-locus sequence typing (MLST) and DNA microarray to determine their antibiotic resistance and virulence profiles, and genetic relatedness.

## Materials and Methods

### MRSA Isolates

The MRSA isolates used in this study were obtained as part of routine diagnostic microbiology investigations. The MRSA were cultured and identified, using traditional diagnostic bacteriological methods including Gram stain, growth on Mannitol Salt Agar, positive DNAse and tube coagulase tests. The isolation and identification of the isolates were performed in the diagnostic microbiology laboratories where initial antibiotic susceptibility testing was also performed with VITEK MS (bioMérieux, Marcy l’Etoile, France). Pure cultures of isolates on blood agar plates were submitted to the Gram-Positive Bacteria Research laboratory, located at the Department of Microbiology, Faculty of Medicine, Kuwait University, where the isolates were retested for purity and preserved in 40% glycerol (v/v in brain heart infusion broth) at −80°C for further analysis. The isolates were recovered by two subcultures on brain heart infusion agar at 35°C before analysis. In total, 5,223 MRSA isolates were received from 13 different hospitals in Kuwait between 1 January, 2016 and 31 December, 2018. DNA microarray analysis performed on the 5,223 isolates revealed that 182 (3.4%) isolates were identified as CC361-MRSA. The 182 CC361-MRSA isolates were investigated further and reported in this study. The 182 CC361 isolates were collected from patients in Adan hospital (*N* = 37; 20.3%), Mubarak hospital (*N* = 32; 17.6%), Sabah hospital (*N* = 23; 12.4%), Maternity hospital (*N* = 20; 10.9%), Al-Amiri hospital (*N* = 19; 10.4%), Al-Razi hospital (*N* = 15; 8.2%), Chest Disease hospital (*N* = 13; 7.1%), Al-Jahra hospital (*N* = 8; 4.4%), Al-Farwaniya hospital (*N* = 8; 4.4%), KOC hospital (*N* = 3; 1.6%), Ibn-Sina hospital (*N* = 2; 1.1%), Dasman Diabetic Centre (*N* = 1; 0.5%) and Army Force hospital (*N* = 1; 0.5%).

### Antibiotic Susceptibility Testing

The CC361-MRSA isolates were retested for susceptibility to antibiotics by the disk diffusion method, and interpreted according to the Clinical Laboratory Standards Institute (Clinical and Laboratory Standard Institute (CLSI), 2015)., The following antibiotic disks obtained from Oxoid (Basingstoke, United Kingdom) were used: Benzyl penicillin (2U), cefoxitin (30 μg), kanamycin (30 μg), mupirocin (200 μg), gentamicin (10 μg), erythromycin (15 μg), clindamycin (2 μg), chloramphenicol (30 μg), tetracycline (10 μg), trimethoprim (2.5 μg), fusidic acid (10 μg), rifampicin (5 μg), ciprofloxacin (5 μg), teicoplanin (30 μg), and linezolid (30 μg). Penicillinase production was tested with the Nitrocefin solution (OXOID-Thermo Scientific) according to the manufacture’s instruction. The minimum inhibitory concentration (MIC) for cefoxitin, vancomycin, teicoplanin, linezolid, and mupirocin were determined using E-test strips (bioMerieux, Marcy l’Etoile, France) and interpreted as described previously CLSI (Clinical and Laboratory Standard Institute (CLSI), 2015). *S. aureus* strains ATCC25923 and ATCC29213 were used as quality control strains for disk diffusion and MIC testing, respectively. Susceptibility to fusidic acid was interpreted according to the British Society to Antimicrobial Chemotherapy (BSAC) (British Society to Antimicrobial Chemotherapy [BSAC], 2013).

### Molecular Typing

#### Staphylococcus Protein A (*spa*) Typing

*Spa* typing was performed using protocol and primers published previously (Harmsen et al., 2003). Bacterial DNA isolation for amplification studies was performed as described previously (Boswihi et al., 2018). Three to five identical colonies of an overnight culture were picked using a sterile loop and suspended in a microfuge tube containing 50 μL of lysostaphin (150 μg/mL) and 10 μL of RNase (10 μg/mL) solution. The tube was incubated at 37°C in the heating block (ThermoMixer, Eppendorf, Hamburg, Germany) for 20 min. To each sample, 50 μL of proteinase K (20 mg/mL) and 150 μL of Tris buffer (0.1 M) were added and mixed by pipetting. The tube was then incubated at 60°C in the water bath (VWR Scientific Co., Shellware Lab, United States) for 10 min. The tube was transferred to a heating block at 95°C for 10 min to inactivate proteinase K activity. Finally, the tube was centrifuged, and the supernatant containing extracted DNA was stored at 4°C till used for PCR.

The PCR protocol consisted of an initial denaturation at 94°C for 4 min, followed by 25 cycles of denaturation at 94°C for 1 min, annealing at 56°C for 1 min, and extension for 3 min at 72°C, and a final cycle with a single extension for 5 min at 72°C. Five μL of the PCR product was analyzed by 1.5% agarose gel electrophoresis to confirm amplification. The amplified PCR product was purified using Micro Elute Cycle-Pure Spin kit (Omega Bio-tek, Inc., United States) and the purified DNA was then used for sequencing PCR. The sequencing PCR product was then purified using Ultra-Sep Dye Terminator Removal kit (Omega Bio-tek, Inc., United States). The Purified DNA was sequenced in an automated 3130x1 genetic analyzer (Applied Biosystem, United States). The sequenced *spa* gene was analyzed using the Ridom Staph Type software (Ridom GmbH, Wurzburg, Germany).

#### DNA Microarray

DNA microarray analysis was performed using the Identibac *S. aureus* genotyping kit 2.0 and the ArrayMate reader (Alere Technology, Jena, Germany) as described previously by Monecke et al. (2011). The DNA microarray analysis was used for the simultaneous detection of SCC*mec* types, antibiotic resistance genotypes and virulence related genes, including PVL, genes encoding species markers, and to allocate clonal complex (CC). *S. aureus* genotyping array is presented in an ArrayStrip format which contains 336 probes printed onto an array located in the bottom of the ArrayStrip. MRSA isolates were grown on blood agar plates at 35°C overnight. DNA extraction of the overnight culture was performed as described by the manufacturer using Identibac *S. aureus* genotyping kit 2.0 (Alere, GmbH, Germany). Linear amplification of the purified DNA was performed in a total of 10 μL of the reaction volume containing 4.9 μL of B1 (labeling reagent), 0.1 μL of B2 (DNA polymerase), and 5 μL of the purified DNA. The PCR protocol consisted of an initial denaturation for 5 min at 96°C, followed by 50 cycles of denaturation for 60 s at 96°C, annealing for 20 s at 50°C, and extension for 40 s at 72°C. hybridization and washing of the labeled arrays were performed as previously described (Monecke et al., 2011). The array was scanned using the ArrayMate reader (CLONDIAG, Alere, Germany) and the image of the arrays was recorded and analyzed using IconoClust software plug-in (CLONDIAG). The result was interpreted as negative, positive, or ambiguous by the software.

#### Multilocus Sequencing Typing (MLST)

MLST was performed for representative isolates belonging to different spa types. The amplification of the seven housekeeping genes was performed using previously described M13-tailed primers (Tan et al., 2006). The amplified targets were sequenced with one pair of M13-tailed primers: 5′-TGTAAAACGACGGCCAGT-3′ and 3′-CAGGAAACAGCTATGACC-5′. The sequencing PCR protocol consisted of initial denaturation for 1 min at 94°C, followed by 25 cycles of denaturation for 10 s at 96°C, annealing at 55°C for 5 s, and extension for 4 min at 66°C. DNA sequencing was performed using a 313091 genetic analyzer (Applied Biosystems, Foster City, CA, United States) in accordance with the manufacturer’s protocol. The sequences were submitted to http://www.pubmlst.net/ where an allelic profile was generated and the sequence type (ST) assigned.

## Results

### Sources of CC361 MRSA Isolates

The isolates were obtained in 2016 (*N* = 55; 30.3%), 2017 (*N* = 56; 30.7%), and 2018 (*N* = 71; 39.0%) from nasal swabs (*N* = 63; 34.6%), wound swabs (*N* = 25; 13.7%), groin swabs (*N* = 11; 6.0%), tracheal aspirates (*N* = 9; 4.9%), pus (*N* = 8; 4.4%), axilla (*N* = 6; 3.3%), ear swab (*N* = 6; 3.3%), high vaginal swab (HVS) (*N* = 6;3.3%), sputum (*N* = 5; 2.7%), skin (*N* = 5; 2.7%), urine (*N* = 5; 2.7%), throat (*N* = 4; 2.2%), blood (*N* = 4; 2.2%), fluid (*N* = 3; 1.6%), and eye swab (*N* = 2; 1.1%). No clinical sources were provided for 20 isolates.

### Molecular Characteristics of the CC361-MRSA Isolates

The isolates belonged to three SCC*mec* types, 22 *spa* types and two sequence types. The distribution of the isolates and their molecular characteristic is presented in Table 1. The SCC*mec* types were type V/VT (*N* = 112; 61.5%), type V (*N* = 34; 18.7 %), and type IV (*N* = 36, 19.8%).

**TABLE 1:** Molecular characteristics of CC361-MRSA isolates from 2016 to 2018.

**Molecular types**	**ST**	**2016**	**2017**	**2018**	**Total**
MRSA Genotypes					
CC361-MRSA [V/VT + fus]		31	29	52	112
*Spa* types					
t3841	672	27	25	34	86
t1309	672	1	1	2	4
t12219	672			1	1
t4336	672		1	1	2
t13751	672			1	1
t14090	672		1		1
t15253	672			1	1
t15778	672	1			1
t17533	672			1	1
t3175	672	1			1
t422	672			1	1
t440	672			1	1
t5357	672			1	1
t779	672			1	1
ND	672	1	1	7	9
CC361-MRSA-[V + fus]		1			1
*Spa* types					
t3841	672	1			1
CC361-MRSA-IV, WA MRSA-29		12	12	12	36
*Spa* types	ST				
t3841	672	7	5	5	17
t11113	672	1			1
t12219	672		1		1
t1309	672	1	2	4	7
t14090	672	1			1
t1427	672		1		1
t311	672			1	1
t3175	672	2	1	1	4
ND	672		2		2
Molecular types	ST	2016	2017	2018	Total
MRSA Genotypes					
CC361-MRSA-V, WA MRSA-70/110		11	15	7	33
*Spa* types					
t315	361	7	9		16
t3841	672	3	3	2	8
t003	672	1			1
t12219	672		1		1
t1309	672			3	3
t17279	672		1		1
t422	672		1		1
t463	361			1	1
t7191	672			1	1
Total		55	56	71	182

*ST, sequence type; ND, not determined.*

The four genotypes were CC361-MRSA [V/VT + fus] (*N* = 112; 61.5%) as the most common genotype, followed by CC361-MRSA-IV, WA MRSA-29 (*N* = 36; 19.8%), CC361-MRSA-V, WA MRSA-70/110 (*N* = 33; 18.1%), and CC361-MRSA-[V + fus] (*N* = 1; 0.5%).

MLST was performed on 58 representative isolates chosen based on *spa* types chosen to include isolates from all clinical samples in all hospitals with the same *spa* types. The MLST identified two closely related sequence types; ST672 (MLST profile: arc-4, aroe-3, glpf-1, gmk-1, pta-11, tpi-72, yqil-11; *N* = 47) and ST361 (MLST profile: arc-4, aroe-3, glpf-1, gmk-1, pta-11, tpi-72, yqil-64; *N* = 11). The sequence types for the rest of the isolates was based on the associated *spa* types. The results showed that most of the isolates belonged to ST672 while 17 isolates belonged to ST361. All CC361-MRSA [V/VT + fus], CC361-MRSA-[V + fus), CC361-MRSA-IV, WA MRSA-29 and 16 of CC361-MRSA-V, WAMRSA −70/110 belonged to ST672 while 17 of CC361-MRSA-V, WAMRSA -70/110 isolates belonged to ST361. Table 1 also shows the yearly distribution of the clones.

The CC361MRSA [V/VT + fus] genotype showed an increasing trend, while the CC361-MRSA-IV, WA MRSA-29 genotype showed a uniform distribution over the three years. In contrast, the prevalence of the CC361-MRSA-V, WA MRSA-70/110 genotype varied by year but declined in 2018.

The 22 *spa* types consisted of t3841 (*N* = 112; 61.5%) as the dominant *spa* type. The other *spa* types were t315 (*N* = 16; 8.8%), t1309 (*N* = 14; 7.7%), t3175 (*N* = 5; 2,7%), t12219 (*N* = 3; 16.5%), t14090 (*N* = 2; 1.1%), t422 (*N* = 2; 1.1%), t4336 (*N* = 2; 1.1%), t003 (*N* = 1; 0.5%), t11113 (*N* = 1; 0.5 %), t13751 (*N* = 1; 0.5%), t1427 (*N* = 1; 0.5%), t15253 (*N* = 1; 0.5%), t15778 (*N* = 1; 0.5%), t17279 (*N* = 1; 0.5%), t17533 (*N* = 1; 0.5 %), t311 (*N* = 1; 0.5%), t440 (*N* = 1; 0.5%), t463 (*N* = 1; 0.5%), t5357 (*N* = 1; 0.5%), t7191 (*N* = 1; 0.5%), t779 (*N* = 1; 0.5%), and 11 isolates (6%) had no determined *spa* type. The dominant spa type, t3841 was distributed among the four genotypes. It constituted the major *spa* type in CC361-MRSA [V/VT + fus] (86/112) and CC361-MRSA-IV, WA MRSA-29 (17/36) but was the second common spa type among the CC361-MRSA-V, WA MRSA-70/110 isolates. The second common spa type, t315 was associated only with the CC361-MRSA-V, WA MRSA-70/110 clone and ST631 (Table 1). The ST631-MRSA-V, WA MRSA-70/110/t315 was detected only in 2016 and 2017.

### Antibiotic Resistance Phenotypes and Genotypes of the CC361-MRSA Isolates

All 182 CC361- MRSA isolates were susceptible to vancomycin and teicoplanin (MIC: ≤2 mg/L) and rifampicin. Besides beta-lactam resistance, resistance was observed to fusidic acid (*N* = 118; 64.8%), kanamycin (*N* = 79; 43.4%), erythromycin (*N* = 66; 36.3%), clindamycin (*N* = 26; 14.3%), gentamicin (*N* = 12; 6.6%), chloramphenicol (*N* = 9; 4.9%), linezolid (MIC: ≥16 mg/L; *N* = 9), trimethoprim (*N* = 5; 2.7%), and tetracycline (*N* = 5; 2.7%). Ten isolates expressed high-level mupirocin resistance. Of the 26 isolates that showed resistance to clindamycin, 15 isolates revealed inducible resistance while 11 isolates expressed constitutive resistance.

The distribution of the antibiotic resistance genes of the CC361-MRSA isolates is presented in Table 2. There was general concordance between resistance phenotypes and genotypes. The penicillin resistance encoding genes *blaZ* and its regulatory genes, *blaI* and *blaR* were found in 177 isolates, while aminoglycoside (gentamicin and kanamycin) encoding genes *aacA-aphD, aphA3*, and *aadD* were detected in 12, 65, and 2 isolates, respectively. The five isolates that were negative for *blaZ*, *blaI*, and *blaR*, were susceptible to penicillin G and negative for penicillinase production but were positive for *mecA* and SCC*mec* genetic elements.

**TABLE 2:** Distribution of antibiotic resistance genes of CC361-MRSA isolates from 2016 to 2018.

**Locus**	**CC361-MRSA [V/VT + fus] (*N* = 112)**	**CC361-MRSA-IV, WA MRSA-29 (*N* = 36)**	**CC361-MRSA-V, WA MRSA-70/110 (*N* = 33)**	**CC361-MRSA-[V + fus] (*N* = 1)**	**Total (*N* = 182)**
Penicillin resistance					
*blaZ*	110	34	32	1	177
*blaI*	110	34	32	1	182
*blaR*	110	34	32	1	182
*mecA*	112	36	3	1	182
MLS-resistance					
*erm*(B)	0	0	1	0	1
*erm*(C)	4	2	16	0	22
*linA*	0	0	1	0	1
*msr*(A)	35	8	4	1	48
*mph(C)*	33	8	4	1	46
*vatA*	0	1	0	0	1
*vatB*	0	1	0	0	1
*vga*(A)	11	1	0	0	12
Aminoglycosides resistance					
*aacA-aphD*	4	2	6	0	12
*aadD*	1	1	0	0	2
*aphA3*	33	10	21	1	65
Trimethoprim resistance					
*dfrS1*	5	0	0	0	5
Fusidic acid resistance					
*fusC*	112	1	2	1	116
*fusB (far1)*	1	1	0	0	2
Tetracycline resistance					
*tet*(K)	3	0	2	0	5
Phenicol resistance					
*Cfr*	0	0	9	0	9
Mupirocin resistance					
*mupA*		0	10	0	10
Quaternary ammonium compound resistance					
*qacA*	1	0	0	0	1
*qacC*	2	0	0	1	3
Streptothricin resistance					
*sat*	33	9	21	1	64
Fosfomycin resistance					
*fosB*	112	36	33	1	182
Locus	CC361-MRSA [V/VT + fus] (*N* = 112)	CC361-MRSA-IV, WA MRSA-29 (*N* = 36)	CC361-MRSA-V, WA MRSA-70/110 (*N* = 33)	CC361-MRSA-[V + fus] (*N* = 1)	Total (*N* = 182)
SCC*mec-*complex associated genes					
*mec*A	112	36	33	1	182
*ugp*Q	112	36	33	1	182
*plsSCC* (COL)	0	0	0	1	1
delta_*mec*R	0	36	0	0	36
*Q9XB68-dcs*	0	36	0	0	36
*ccr*C	112	0	33	1	146
*ccr*B-2	0	36	0	0	36
*ccr*A-2	0	36	0	0	36
*ccr*A-3	0	1	0	0	1
*ccr*AA	112	0	33	1	146

*blaZ, beta-lactamase gene; blaI, beta lactamase repressor (inhibitor); blaR, beta-lactamase regulatory protein; mecA, penicillin binding protein 2; erm(B) + erm(C), rRNA methyltransferase associated with macrolide/lincosamide resistance; linA, lincosaminide nucleotidyltransferase; msr(A), macrolide efflux pump; mph(C), macrolide phosphotransferase II; vatA, virginiamycin A acetyltransferase; vatB, acetyltransferase inactivating streptogramin A; vga(A), ABC transporter conferring resistance to streptogramin A; aacA-aphD, aminoglycoside adenyl-/phosphotransferase (gentamicin, tobramycin); aadD, aminoglycoside adenyltransferase (neomycin, kanamycin, and tobramycin); aphA3, aminoglycoside phosphotransferase (neomycin, kanamycin); dfrS1, dihydrofolate reductase mediating trimethoprim resistance; fusC + fusB (far1), fusidic acid resistance gene; tet(K), tetracycline efflux protein; Cfr, 23S rRNA methyltransferase; mupA, isoleucyl-tRNA synthetase; qacA, multidrug efflux protein A; qacC, multidrug efflux protein C; sat, streptothricin acetyltransferase; fosB, metallothiol transferase; ugpQ, glycerophosphoryl diester phosphodiesterase (associated with mecA); plsSCC (COL), plasmin-sensitive surface protein; delta_mecR, truncated signal transducer protein MecR1; Q9XB68-dcs, hypothetical protein from SCCmec elements; ccrC, cassette chromosome recombinase gene C; ccrB-2, cassette chromosome recombinase gene B-2; ccrA-2, cassette chromosome recombinase gene A-2; ccrA-3, cassette chromosome recombinase gene A-3; ccrAA, Putative protein homologue to cassette chromosome recombinase A genes.*

Of the 66 erythromycin-resistant isolates, 22 isolates carried *ermC*, one isolate carried *ermB*. While 48 and 46 isolates carried *msrA* and *mphC* encoding genes, respectively, *mefA* (macrolide efflux protein A), *vatA* (virginiamycin A acetyltransferase), *vatB* (acetyltransferase inactivating streptogramin A) and *linA* (lincosaminide nucleotidyltransferase) were detected sporadically among the CC361 isolates. Two isolates harbored *fusB* (*far1*) gene. Tetracycline resistance gene *tetK*, was detected in five isolates, while trimethoprim resistance gene *dfrS1* was also found in five isolates.

Whereas all of the CC361-MRSA [V/VT + fus) isolates were resistant to fusidic acid mediated by *fusC*, the CC361-MRRSA-V-WA-MRSA-70/110 isolates were more resistant to erythromycin mediated by erm(C), high-level mupirocin resistance mediated by *mupA*, and chloramphenicol and linezolid mediated by *cfr*.

### Prevalence of Virulence-Related Genes

The prevalence of the virulence factors amongst the CC361 MRSA isolates are presented in Table 3. All 182 isolates were positive for genes encoding the accessory gene regulator type I (*agr*I) and capsular polysaccharide type 8 (*cap*8) but lacked genes for Panton Valentine Leukocidin (PVL).

**TABLE 3:** Virulence factors amongst the CC361-MRSA strains.

**Locus**	**CC361-MRSA [V/VT + Fus] (*N* = 112)**	**CC361-MRSA-IV, WA MRSA-29 (*N* = 36)**	**CC361-MRSA-V, WA MRSA-70/110 (*N* = 33)**	**CC361-MRSA-[V + fus] (*N* = 1)**	**Total (*N* = 182)**
Enterotoxins					
*sea*	1	2	3	0	6
*seb*	1	7	0	0	8
*sec*	2	0	17	0	19
*sed*	2	1	0	0	3
*see*	0	1	0	0	1
*sel*	2	0	18	0	20
*sek*	0	7	0	0	7
*seq*	0	7	0	0	7
*egc gene cluster	112	36	33	1	182
Toxic shock syndrome toxin-1 (TSST-1)					
*tst1*	2	0	17	0	19
Lukocidins					
Luk-PV (P83)	12	1	0	0	13
lukF	112	36	33	1	182
lukS	112	36	33	1	182
lukD	112	36	33	1	182
lukE	112	36	33	1	182
lukX	109	36	32	1	178
lukY	111	36	31	1	180
Immune Evasion Cluster (IEC)					
*chp-scn-sak* (Type B)	2	2	16	0	20
*scn-sak* (Type E)	102	32	17	1	152
Negative for IEC genes	8	2	-	-	10
Hemolysins					
*hlgA*	111	36	33	1	182
*Hla*	110	35	26	1	172
*Hlb*	111	36	33	1	181
*Hl*	112	36	33	1	182
*hlIII*	112	36	33	1	182
Adhesion factors					
*clfA*	112	36	33	1	182
*clfB*	112	36	33	1	182
*fnbA*	112	36	33	1	182
*fnbB*	112	36	33	1	182
*sasG*	112	36	33	1	182
*Map*	112	36	33	1	182
*cna*	0	0	0	0	0

*egc gene cluster: seg, sei, selm, seln, selo, selu. hlgA, hemolysin gamma; hla, hemolysin alpha; hlb, hemolysin beta; hl/hlIII, putative membrane protein; sak, staphylokinase; chp, chemotaxis-inhibiting protein; scn, staphylococcal complement inhibitor, clfA, clumping factor A; clfB, clumping factor B; fnbA, fibronectin-binding protein A; fnbB, fibronectin-binding protein B; sasG, *Staphylococcus aureus* surface protein G; map, major histocompatibility complex class II; can, collagen-binding adhesin.

The most common enterotoxin gene, egc gene cluster (*seg, sei, selm, seln, selo*, and *selu*), was detected in all 182 CC361 isolates. This was followed by *sel* (*N* = 20; 11%) and *sec* (*N* = 19; 10.4%). Genes for Toxic shock-syndrome toxin (*tst1*) was detected in 19 isolates. The *sec* and *sel* were more common in the CC361-MRSA-V WA MRSA-70/110 isolates. Similarly, 17 of the 19 tst1-positive isolates belonged to CC361-MRSA-V WA MRSA-70/110. Only two of the CC361-MRSA [V/VT + fus] isolates harbored the *tst1*. On the other hand, *sek* and *seq* genes were common among the CC361-MRSA-V, WA MRSA-29 isolates (Table 3).

The isolates varied in the carriage of genes for the immune evasion cluster (IEC). Twenty isolates carried immune evasion cluster genes of type B (*scn*, *chp*, and *sak*), whilst 152 isolates carried the immune evasion cluster genes of genes for type E (*scn* and *sak*). Ten isolates were negative for the IEC genes.

The hemolysin encoding genes *hlgA*, *hl*, and *hlIII*, were detected in all 182 isolates. However, *hlb* was detected in 181 of the 182 isolates while *hla* was detected in 172 isolates (94.5%). In addition, all 182 isolates were positive for genes encoding clumping factors A and B (*clfA* and *clfB*), fibronectin-binding proteins A and B (*fnbA* and *fnbB*), *Staphylococcus aureus* surface protein G (*sasG*) and major histocompatibility complex class II analog protein (*map*). All isolates lacked *cna* that codes for the collagen-binding adhesin.

## Discussion

This study has demonstrated a steady expansion of the CC361-MRSA lineage among patients in Kuwait hospitals in recent years. Until recently, CC361-MRSA was reported sporadically from human patients in Oman (Udo et al., 2014), Abu Dhabi (Weber et al., 2010), Australia (Monecke et al., 2011), Ireland (Kinnevey et al., 2014), Bangladesh (Afroz et al., 2008), Kuwait (Boswihi et al., 2016), Saudi Arabia (Senok et al., 2019), and Switzerland (Etter et al., 2020) as well as in monkeys in Nepal (Roberts et al., 2019), in cattle in Czech Republic (Tegegne et al., 2017), and in ready-to-eat food in Bangladesh (Islam et al., 2019). However, this report represents the largest number of CC361-MRSA reported to date. The number of CC361-MRSA obtained from human patients increased from two isolates identified for the first time in Kuwait in 2010 (Boswihi et al., 2016), to 55 in 2016, 56 in 2017 and 71 in 2018. Similarly, increases in the proportion of CC361-MRSA in human patients have been observed in the UAE and Saudi Arabia (Senok et al., 2020), making the CC361-MRSA no longer a rare but an evolving clone in the Arabian Gulf region.

The isolates were obtained from different clinical samples including wound swabs, nasal swabs, blood culture, vaginal swabs, sputum, tracheal aspirates, and urine highlighting the capacity of the CC361-MRSA isolates to colonize or cause superficial as well as invasive infections.

DNA microarray analysis revealed different genotypes dominated by CC361-MRSA [V/VT + Fus] which constituted 61.5% of the isolates. The other genotypes detected were CC361-MRSA-V WA MRSA-29 (*N* = 36; 19.8%), CC361-MRSA-V WA MRSA-70/110 (*N* = 33; 18.1%) and CC361-MRSA-[V + fus] variant (*N* = 1; 0.5%). Although there were no significant differences in the distribution of the four CC361 genotypes from 2016 to 2018, the CC361-MRSA [V/VT + fus] isolates increased from 31 in 2016 to 71 in 2018 (Table 1) suggesting its higher transmission capacity. The acquisition of the variant V/VT genetic element represents an evolutionary event that probably confers greater capacity to spread. Although this clone appears to be restricted to the countries of the Gulf at this time, it has the potential to spread widely as the highly virulent USA300 MRSA clone that emerged initially as a less virulent and less resistant clone (Strauß et al., 2017). The USA300 MRSA clone gradually acquired multiple antibiotic resistance, SCC*mec* Iva genetic element, genes for PVL, arginine catabolic mobile element, and a specific mutation in capsular polysaccharide gene, *capSE* and became highly transmissible following its introduction to North America from Central Europe (Strauß et al., 2017).

Despite the smaller numbers reported in Saudi Arabia (8 0f 12 isolates) (Senok et al., 2019) and the UAE (20 of 35 isolates) (Senok et al., 2020) compared to those in Kuwait, CC361-MRSA [V/VT + fus] isolates were also the most common strain type reported in these countries. Similarly, the second common genotype in this study, CC361-MRSA-IV, WA-MRSA 29, was also the second most common CC361-MRSA in the UAE (Senok et al., 2020), suggesting similar trends in the evolution of the CC361-MRSA in these countries.

MLST identified two closely related sequence types; ST672 (slv of ST361) and ST361 among the four different strain types, while *spa* typing revealed 22 spa types. Most of the isolates (112/182) belonged to ST672/t3841 (Table 1) followed by ST361/t315 (16/182). We observed that whereas ST672/t3841 isolates were found in all four strain types, ST361/t315 isolates were only detected among CC361-MRSA-V, WA MRSA-70/110 strain type that were isolated only in 2016 and 2017. It is interesting that one of the two CC361 – MRSA isolates that were isolated in Kuwait in 2010 was ST361-MRSA-IV/t315 (Boswihi et al., 2016) whereas the current isolates are ST361-MRSA-V/t315 suggesting that the current isolates were acquired independently, and did not evolve from the 2010 isolate. Other studies have reported CC361-MRSA strains as belonging to ST361-MRSA-IV (Afroz et al., 2008; Coombs et al., 2011; Kinnevey et al., 2014; Al-Zahrani et al., 2019) or ST672-MRSA-V (Coombs et al., 2011; Shambat et al., 2012; Balakuntla et al., 2014; Santosaningsih et al., 2016; Sunagar et al., 2016). ST361-MRSA/t315 isolates have also been reported from processed fish fingers and Chapatti in Dhaka, Bangladesh (Islam et al., 2019) probably because of human contamination of the ready-to-eat foods.

The CC361-MRSA strains appear to have appeared independently in the GCC countries. It was first reported in Abu Dhabi, UAE, from patients in 2009 (Weber et al., 2010) followed by the report in two patients in Kuwait in 2010 (Boswihi et al., 2016) and in two patients in Oman in 2011 (Udo et al., 2014). However, prior to their emergence in the GCC countries, an isolate of ST361-MRSA-IV was isolated from a patient in Bangladesh in 2004 (Afroz et al., 2008) and two isolates of ST672-MRSA-V were reported among MRSA isolates obtained in 2004–2006 in India (Shambat et al., 2012) supporting the recent emergence of CC361-MRSA in the GCC countries.

The isolates belonged to 22 different *spa* types with t3841 (*N* = 112; 61.51%), t315 (*N* = 16; 8.8%), and t1309 (*N* = 14; 7.7%) constituting 78 percent of the isolates. Whereas t3841 and t1309 were associated with ST672 distributed in three strain types, t315 was associated only with ST361 in a single strain type, CC361-MRSA-V, WA MRSA 70/110, in this study. Similarly, t315 has been associated only with ST361 in studies conducted in Australia (Coombs et al., 2011), Ireland (Kinnevey et al., 2014), Austria (Zarfel et al., 2016), and from cattle in Czech Republic (Tegegne et al., 2017) where *spa* typing were also reported. In contrast, ST672 isolates were associated with t1309 in studies that reported *spa* types in Australia (Coombs et al., 2011) and India (Shambat et al., 2012). These studies support the recent acquisition of the ST672-MRSA [V/VT + Fus]/t3841 strain in Kuwait. The origin of the dominant t3841 isolates in this study is not clear since previous ST672 isolates have largely been associated with t1309 (Coombs et al., 2011; Shambat et al., 2012). However, a previous study in Kuwait detected t3841 among MSSA isolates in the country (Vali et al., 2017). It is possible that the t3841 MRSA isolates in this study emerged from a locally circulating MSSA isolate that acquired *mecA*. An isolate of t3841 MSSA had also been reported in India (Shambat et al., 2012).

Besides the diversity in *spa t*ypes, the insolates in this study harbored different SCC*mec* types consisting of SCC*mec* types IV, V and V/VT that are usually associated with community-associated genotypes. Apart from SCC*mec* V/VT, SCC*mec* types IV and V were also reported in CC361-MRSA from human patients in Western Australia (Monecke et al., 2011), Ireland (Kinnevey et al., 2014) and Bangladesh (Afroz et al., 2008), Abu Dhabi (Weber et al., 2010), Oman (Udo et al., 2014) and Saudi Arabia (Senok et al., 2019) and in animals in Nepal (Roberts et al., 2019) and Czech Republic (Tegegne et al., 2017). CC361 isolates were also reported to harbor SCC*mec* VIII in Australia where it is known as WA MRSA-28 (Nimmo and Coombs, 2008). These observations demonstrate the ability of CC361 to acquire different SCC*mec* elements. The detection of SCC*mec* V/VT together with *spa* type t3841 only in the current isolates supports the recent emergence of these strains in the GCC countries.

The CC361-MRSA isolates were resistant to different antibiotics including gentamicin, kanamycin, erythromycin, clindamycin, tetracycline, trimethoprim fusidic acid and high-level mupirocin and harbored *aacA-aphD*, *aphA3*, *msrA*, *tet(K)*, *dfrS1*, *fusC*, and *mupA* mediating resistance to the corresponding antibiotics as has been reported in isolates from Saudi Arabia (Senok et al., 2019), Abu Dhabi (Monecke et al., 2011), and Australia (Coombs et al., 2011). All the 112 ST672-MRSA-[V/VT + fus] isolates harbored *fusC* and was responsible for the high prevalence of fusidic acid resistance in this study. Fusidic acid resistance mediated by *fusC* was also reported in CC361-MRSA isolates obtained from dental room environment and human patients in Saudi Arabia (Senok et al., 2019). Fusidic acid resistance has remained a major problem in MRSA isolates in Kuwait for some years, and has been suggested to be due to independent acquisition of fusidic acid determinants in isolates belonging to diverse backgrounds, and the consumption of over the counter fusidic acid preparations which are available without prescription in the country (Boswihi et al., 2018).

We detected linezolid resistance in nine CC361-MRSA-V, WA MRSA-70/110 isolates that were resistant to chloramphenicol and linezolid, and were positive for *cfr* that encodes resistance to linezolid, chloramphenicol, lincosamides, and streptogramin A (Pillai et al., 2002; Long et al., 2006; Morales et al., 2010). This is the first report of linezolid resistance in *S. aureus* in Kuwait. Linezolid resistance mediated by *cfr* was recently reported in a recent MRSA isolate obtained in the United Arab Emirate (Senok et al., 2020) indicating that linezolid resistance is emerging in these countries and should raise awareness of an emerging problem. In contrast, none of the CC361-MRSA-V, WA MRSA-70 reported in Australia expressed resistance to linezolid or high-level mupirocin (Coombs et al., 2011). Since its approval for clinical use, Linezolid has remained an important treatment option for treating infections caused by MRSA and glycopeptide-resistant enterococci (Pillai et al., 2002). Therefore, the emergence of linezolid resistance in MRSA observed in this study is concerning because it will limit treatment options available for MRSA infections. The 10 high-level mupirocin-resistant isolates harbored *mupA* that codes for this resistance. The *mupA* mediated high-level mupirocin resistance have been previously in MRSA belonging to other genetic backgrounds in Kuwait (Udo et al., 2001; Udo and Sarkhoo, 2010).

The CC361-MRSA isolates in this study were negative for genes encoding PVL similar to CC631-MRSA isolates reported previously in Oman (Udo et al., 2014), UAE (Senok et al., 2020), Australia (Coombs et al., 2011), and most of the isolates in Saudi Arabia (Senok et al., 2019). However, two isolates consisting of, an isolate of CC361-MRSA-V/VT (PVL+) isolated in Saudi Arabia (Senok et al., 2019) and an isolate of ST361-MRSA-/t315 isolated from ready-to-eat food sample in Bangladesh (Islam et al., 2019), were positive for PVL suggesting that PVL is rare among CC361-MRSA isolates.

The isolates were all positive for agr type 1 (*agr1*) and capsular polysaccharide type 8 (*cap8*), egc gene cluster (*seg*, *sei*, *selm*, *seln*, *selo*, and *selu*), hemolysins genes *hlgA*, *Hl*, *hlIII* and adhesion factor genes *clfA*, *clfB*, *fnbA*, *fnbB*, *sasG*, and *Map*. *clfA*, *clfB*, *fnbA*, and *fnbB* and were negative for *cna* that encodes collagen binding adhesin, as have also been reported previously for other CC361-MRSA isolates (Afroz et al., 2008; Coombs et al., 2011; Monecke et al., 2011; Shambat et al., 2012; Boswihi et al., 2016; Senok et al., 2019) indicating that these are constitutional characteristics of CC361 isolates. In addition, the CC361-MRSA isolates in this study varied in the carriage of enterotoxin genes, *sea*, *seb*, *sec*, *sed*, *sel*, *sek*, and *seq* similar to the reports of studies on prevalence of enterotoxins genes in CC361-MRSA isolates in several countries (Boswihi et al., 2018; Senok et al., 2019, 2020; Etter et al., 2020) including CC361-MRSA obtained from monkeys and ready-to-eat food (Islam et al., 2019; Roberts et al., 2019).

Only 19 of the 182 isolates, consisting of 17 CC361-MRSA-V, WA MRSA-70/110, and two CC361-MRSA [V/VT + Fus], were positive for *tst1* in this study. Similarly, small numbers of C361 MRSA carrying the *tst1* gene were in Bangladesh (Islam et al., 2019) and in monkeys in Nepal (Roberts et al., 2019).

Most of the CC361- MRSA isolates in this study harbored type E (*scn*, *sak*) (*N* = 152; 83.5%) or type B (*scn*, *chp*, and *sak*) (*N* = 20; 11.0%) immune evasion cluster genes. Likewise, CC361-MRSA reported in Australia (Coombs et al., 2011) Saudi Arabia (Senok et al., 2019), and United Arab Emirates (Senok et al., 2020) also carry either type E or type B immune evasion cluster (IEC) genes. The immune evasion cluster genes binds specifically with compounds of the human innate immune system to protect bacteria from the human innate immune system (Resch et al., 2013; Sieber et al., 2020) and are therefore used to distinguish *S. aureus* of human from those of animal origin since human isolates harbor the IEC genes (Resch et al., 2013). The presence of IEC carrying *S. aureus* in animals usually suggests human contamination (Resch et al., 2013; Sieber et al., 2020). Viewed from this perspective, the presence of type E (*scn* and *sak*) IEC in CC361- MRSA that were recovered from monkeys (Roberts et al., 2019) which is similar to the IEC content of 94.5% of our isolates may suggest a human origin for the monkey isolates. Although CC361-MRSA have also been isolated from cattle (Tegegne et al., 2017), and ready to-eat-food (Islam et al., 2019) the IEC genes contents of these isolates were not reported. Hence it is not possible to speculate their origin. Ten of the isolates in this study consisting of five ST672-MRSA-V/VT + Fus/t3841 and five ST672-MRSA-IV, WAMRSA 29/t14690/t14271/t1309/t3175/3841 were negative for IEC genes suggesting the loss of the bacteriophage that bears the IEC genes.

## Conclusion

In conclusion, this study reports an increase in the prevalence of the CC361-MRSA isolates with the dominance and transmission of a newly emerged ST672-MRSA [V/VT + fus] genotype in Kuwait hospitals. The CC361-MRSA isolates expressed resistance to different antibiotics including linezolid resistance observed for the first time in Kuwait. The isolates were negative for genes encoding PVL but harbored common enterotoxins encoding genes (e.g., *egc* gene cluster). The detection of the various virulence genes in these isolates and their isolation from different clinical samples indicate their capacity to cause serious infections like other virulent MRSA lineages. Continuous surveillance is necessary to monitor and evaluate emerging MRSA clones in Kuwait to assist in developing better means of prevention and management.

## Data Availability Statement

The original contributions presented in the study are included in the article/supplementary material, further inquiries can be directed to the corresponding author/s.

## Author Contributions

SB carried out the laboratory work. ES, SM, EM, and RE performed the data analysis. EU performed the experimental design. ES, SM, SB, and EU carried out the manuscript writing and editing. All authors read and approved the final manuscript.

## Conflict of Interest

The authors declare that the research was conducted in the absence of any commercial or financial relationships that could be construed as a potential conflict of interest.
